# Primary Vaginal Chloroma: A Rare Case Report

**Published:** 2018-07-01

**Authors:** Irappa Madabhavi, Apurva Patel, Mitul Modi, Swaroop Revannasiddaiah, Chidanand Chavan

**Affiliations:** 1 Department of Medical and Paediatric Oncology, Kerudi Cancer Hospital, Bagalkot, Karnataka, India; 2 Department of Medical and Paediatric Oncology, Gujarat Cancer and Research Institute, Ahmedabad, Gujarat, India; 3 Department of Pathology, Gujarat Cancer and Research Institute, Ahmedabad, Gujarat, India; 4 Department of Radiotherapy, Government Medical College, Haldwani, India

**Keywords:** Chloroma, Myeloid sarcoma, Patient, Decitabine

## Abstract

Chloroma (granulocytic sarcoma or myeloid sarcoma) is a rare malignant extra-medullary neoplasm of myeloid precursor cells. It is usually associated with myeloproliferative disorders but very rarely may precede the onset of leukaemia. Here we are presenting a rare case of chloroma in a female patient without initial presentation of AML. 38 year old female patient, with performance score-1 had complaining of per vaginal bleeding for 1-2 days. Patient consulted gynaecologist and underwent biopsy from anterior fornix of vagina. Biopsy material was positive for LCA (leukocyte common antigen), MPO (myeloperoxidase), c-kit positive on IHC (immunohistocytochemistry) while negative for cytokeratin, synaptophysin, chromogranin, CD20 (cluster of differentiation. Whole body CT scan was non informative except mass lesion at vagina. Patient was given 3+7 induction chemotherapy which was tolerated well followed by high dose cytarabine as consolidation therapy.

## Introduction

 Chloroma (also known as myeloid sarcoma, granulocytic sarcoma) is a rare EM (extramedullary) tumour of immature myeloid cells. Isolated chloroma, defined by the absence of a history of leukemia, myelodysplastic syndrome (MDS) or myeloproliferative neoplasm and a negative bone morrow biopsy, has been described in limited case reports. We are reporting here a case of chloroma in a female geriatric patient without initial presentation of acute myeloid leukemia (AML). The patient received decitabine and showed a partial response to chemotherapy.

## Case presentation

A 38-year-old female (gravid 3, Para 3) with performance status of 1 had a 20-day complaint of irregular vaginal bleeding. A biopsy of the anterior vaginal fornix revealed small cell endocrine tumour. The patient was then referred to our center for further management. 

The patient was first seen by our specialist gynec onco surgeon. On examination, she was in perfect physical condition, without any history of weight loss, fever, or night sweats. Pelvic examination suggested polypoid tumor mass, 4.5 X 4 X 2 cm, arising from lateral fornix of right side of vagina. Biopsy material showed chloroma. LCA, MPO (myeloperoxidase) and c-kit were positive on IHC (immunohistochemistry), while negative for cytokeratin, synaptophysin, chromogranin, CD20, CD99 and CD79a. Complete hemogram with peripheral smear, renal and liver function test, blood sugar, lactose dehydrogenase, uric acid were normal. There was no palpable organomegaly and lymphadenopathy. Contrast enhanced Computer Tomography scan of thorax, abdomen and pelvis showed 5 x 5 cm mass lesion lateral to cervix, supra diaphragmatic lymphadenopathy, infra diaphragmatic lymphadenopathy, mild hepatomegaly, and splenomegaly. Bone marrow aspiration, trephine biopsy, conventional cytogenetic analysis and 2D echo were also normal.

Disease diagnosis and chemotherapy side effects were explained to the patient. After written consent, the patient received induction chemotherapy with the “3+7” regimen, including daunorubicin and cytarabine. The patient showed a partial response after completion of chemotherapy, and then she was consolidated with high-dose cytarabine and local radiotherapy. She was asymptomatic and was kept under regular surveillance for 12 months at our institution.

## Discussion

 AML may present in a variety of EM tissues with or without bone marrow disease. EM involvement by AML is relatively rare, but clinically often poses diagnostic challenge and therapeutic dilemma. It was first described in 1811 and later named “chloroma” by King in 1853 because of its green colour caused by the presence of myeloperoxidase (MPO) ^1^.

Chloroma is reported in 2.5%-9.1%^2^ of patients with AML and occurs concomitantly, following or rarely, antedating the onset of systemic bone marrow leukaemia. Certain known AM cytogenetic abnormalities like t (8, 21) have been associated with a higher incidence. In our case, there was not any translocation. Chloroma can also develop at relapse with or without marrow involvement. The frequency with which certain chloroma sites are accompanied by marrow involvement has not been adequately studied. Clinical manifestations are varied, depending on the various sites and sizes at presentation. The most common locations include the soft tissue, bone, periosteum and lymph nodes. Clinically significant involvement of the female genital tract is rare. The most commonly involved organ is the ovary estimated at 36.4%, followed by the cervix and uterus^3^. Vaginal involvement is very unusual as in our case. Most of the patients (81–83%) present with vaginal bleeding same as in our case ^4^. 

The diagnosis is not always easy when chloroma appears at an EM site, especially in the absence of AML. Most of them are poorly differentiated, and only in 44% of cases the correct diagnosis is made or suspected. The most common misdiagnosis is the high-grade non-Hodgkin’s lymphoma. Both are composed of diffusely infiltrating, discohesive cells that tend to spare normal structures, and which may contain scattered lymphocytes. In chloroma, however, the nuclei are typically slightly smaller with more finely dispersed chromatin, and some cells may show recognizable myeloid differentiation. The immunohistochemical stains are often required to confirm the diagnosis. 

Treatment of isolated chloroma is variable and often delayed. The treatment options include local radiotherapy and systemic chemotherapy. The role of radiotherapy remains controversial in post- remission therapy. When vital organs are involved, local RT can be used to hasten control of symptoms. Few reports of complete remission have been described after aggressive multimodality treatment of chloroma in other sites, including disseminated chloroma without evidence of AML. Because of the toxicity and uncertain benefit of standard induction chemotherapy in the older population of patients with AML, many of whom are offered only supportive care or low- intensity chemotherapy ^6^^, ^^7^. Data suggestive of more mortality in patients with poor performance status when conventional cytotoxic drugs were used. It appears appropriate to treat chloroma with AML-type chemotherapy protocols even in the absence of systemic manifestations since acute myeloid leukemia is almost always present. As our patient was young and had good performance status, she was offered high-dose induction chemotherapy, followed by consolidation therapy.

Lan TY et al. studied 24 patients with chloroma and found a 5-year survival rate of approximately 20% in patients with chloroma. In this study, patients undergoing chemotherapy alone had a significantly longer survival time (p-value= 0.0009) compared to those undergoing chemotherapy combined with radiation or surgery^5^.

Phase II and II studies have demonstrated that decitabine ^8^^, ^^9^, has significant activity in the treatment of AML over a wide range of doses and in a variety of administration schedules*. *Additional investigation will be required to define the best use of decitabine in the treatment of elderly patients with AML. Kantarjian et al. studied (phase III) decitabine in elderly population and observed that the adverse effects of cytarabine and decitabine are the same. It is concluded that decitabine improves response rate compared with standard therapies without major differences in safety.

## CONCLUSION

 Chloroma involving vagina in an elderly patient without AML is a highly unusual presentation and remains a diagnostic and therapeutic challenge. Immunochemistry staining and pathology are often required to confirm disease diagnosis. Treatment consists of induction chemotherapy according to general condition of the patient. The role of local RT as consolidation is still controversial.
